# Integrated mental health treatment and employment services for refugees with PTSD: randomised controlled trial

**DOI:** 10.1192/bjo.2026.12070

**Published:** 2026-08-05

**Authors:** Maja Bruhn, Henriette Laugesen Attardo, Hinuga Sandahl, Lene Falgaard Eplov, Carsten Hjorthøj, Jessica Carlsson

**Affiliations:** Department of Clinical Medicine, Faculty of Health and Medical Sciences, University of Copenhagen, Copenhagen, Denmark; Competence Centre for Transcultural Psychiatry, Mental Health Centre Ballerup, Copenhagen University Hospital – Herlev and Gentofte Hospital, Copenhagen, Denmark; Copenhagen Research Unit for Recovery, Mental Health Centre Amager, Copenhagen University Hospital – Amager and Hvidovre Hospital, Copenhagen, Denmark; Copenhagen Research Center for Mental Health – CORE, Mental Health Centre Copenhagen, Copenhagen University Hospital, Copenhagen, Denmark; Department of Public Health, Section of Epidemiology, University of Copenhagen, Copenhagen, Denmark

**Keywords:** Trauma and stressor-related disorders, transcultural psychiatry, randomised controlled trial, psychosocial interventions, service development

## Abstract

**Background:**

Post-migration stressors can exacerbate post-traumatic stress disorder (PTSD) and reduce treatment effectiveness among refugees. Evidence for integrated care models in high-income settings remains limited.

**Aims:**

To compare treatment as usual (TAU) with an add-on integrated care intervention for unemployed refugees with PTSD.

**Method:**

We conducted a two-arm, parallel-group superiority trial with 1:1 randomisation to TAU or TAU with an add-on integrated care intervention, delivered at a specialised out-patient clinic in Denmark (ClinicalTrials.gov NCT04244864). TAU included sessions with a psychologist and physician over 8–12 months. The integrated care intervention also included structured collaboration with employment services. The primary outcome was functioning, using the 12-item World Health Organization Disability Assessment Schedule 2.0 (WHODAS) interview. Secondary outcomes included symptoms, quality of life and post-migration stressors. Analyses followed the intention-to-treat principle, using analysis of covariance and linear regression with multiple imputations.

**Results:**

The study included 195 patients in treatment from 2020 to 2025. No difference was observed in WHODAS score between groups pre- to post-treatment (mean difference 0.30, 95% CI −2.40 to 3.00; *P* = 0.825). Similarly, no differences were found for secondary or exploratory outcomes, and overall change was limited. However, the integrated care group had a lower rate of early dropout (*P* = 0.042) and higher level of treatment satisfaction (*P* = 0.035).

**Conclusions:**

Integrated care was feasible but not superior to TAU in improving outcomes for refugees with longstanding symptoms and unemployment. Future research should examine how the timing and intensity of integrated care interventions influence outcomes, including earlier implementation and adequate support for refugees with longstanding and complex needs.

In 2024, the number of forcibly displaced people reached an unprecedented 123 million worldwide; this included 43 million refugees, of whom a quarter resettle in high-income countries.^1^ Refugees are at high risk of mental disorders, particularly post-traumatic stress disorder (PTSD) and depression, with prevalence estimates of around 30%.^2^ Although evidence-based psychosocial treatments for PTSD can be effective for refugees in high-income settings, treatment response is heterogeneous, and a substantial proportion of individuals show limited improvement.^3^ A key explanation is the impact of social determinants of mental health after resettlement, often described in the refugee literature as post-migration stressors. These include unemployment, poverty, insecure legal status, family separation and social isolation, all of which can maintain or exacerbate symptoms of mental disorders.^4,5^ In Denmark, refugees granted residence have access to the labour market and, when unemployed, are generally affiliated with municipal employment services as part of the employment and benefit system. Employment, social and healthcare services are organised across separate sectors with differing mandates and priorities.; this may hinder coordination and leave people with multifaceted health and social needs to navigate multiple agencies.^6^ In combination, post-migration stressors and fragmented services may limit the effectiveness of mental health treatment for refugees.^7–11^ Integrated care models have been developed in other psychiatric populations to bridge health and welfare systems, reduce fragmentation and address social determinants that impede recovery, and these are now widely promoted in mental healthcare.^12–15^ However, refugees undergoing mental health treatment have remained largely absent from this research base.^7^ Despite recognition that social determinants may undermine treatment,^8,10,11,16^ no randomised controlled trials (RCTs) have tested integrated care models that simultaneously address mental health and post-migration stressors among refugees in high-income settings.^7^ Such trials in real-world settings are needed to determine whether adapting standard PTSD treatment to incorporate targeted support for post-migration stressors can improve outcomes.^7,9,17^

To address this gap, we developed an integrated care intervention that embedded systematic collaboration with employment services into specialised mental health treatment for unemployed refugees with PTSD. As many post-migration stressors influencing recovery extend beyond the scope of specialised mental health services, the intervention was designed to facilitate coordinated action across sectors. It was expected that addressing these stressors would support improvements in both mental health and functioning. The aim of the present RCT was to evaluate the effects of an add-on integrated care intervention compared with treatment as usual (TAU) for unemployed refugees with PTSD, with respect to measures of functioning, mental health symptoms, quality of life and post-migration stressors. We hypothesised that participants receiving the add-on integrated care intervention would have superior outcomes across all measures compared with those receiving TAU.

## Method

### Trial design and setting

The study was a pragmatic, parallel-group, two-arm superiority trial with a 1:1 allocation ratio to either TAU or TAU with an add-on integrated care intervention. The trial formed part of a larger mixed-methods research programme, which also included a separate qualitative study exploring patient and provider experiences, a health economic evaluation, and a follow-up study using clinical and registry data. The setting was a highly specialised mental health out-patient clinic for refugees and migrants, the Competence Centre for Transcultural Psychiatry (CTP), in the Capital Region of Denmark. This clinic serves a catchment area of nearly two million people, and culturally sensitive care, including use of interpreters and attention to patients’ cultural and social context, is an integral part of routine clinical practice. The intersectoral collaboration involved five municipalities, representing about half of the catchment area. Participants were randomised electronically, stratified by municipality, using a sequence generated by an independent researcher.

The authors assert that all procedures contributing to this work comply with the ethical standards of the relevant national and institutional committees on human experimentation and with the Helsinki Declaration of 1975, as revised in 2013. All procedures involving patients were reviewed by the Regional Ethics Committee for the Capital Region of Denmark, which waived the need for ethical approval upon review of the protocol (journal no. H-19067136). The trial was approved by the Danish Data Protection Agency (P-2019-327) and registered at ClinicalTrials.gov (NCT04244864) before enrolment on 27 January 2020. The full statistical analysis plan was published before trial completion. The trial protocol, including a detailed description of the study design and methods, has also been published.^18^

### Participants

Patients were referred to the CTP by their general practitioner, a privately practising psychiatrist, or a physician from a mental health or somatic hospital, all within the publicly funded Danish healthcare system. In an initial assessment, a physician assessed RCT eligibility criteria, and all referred patients who were interested in treatment and met the criteria were invited to participate in the study. Table 1 provides eligibility criteria. All participants provided written informed consent before randomisation and were free to withdraw from the study at any time. The physician collected baseline information on trauma history, migration experiences, post-migration circumstances, and somatic and psychiatric medical history, as well as performing a clinical and diagnostic assessment. Standardised diagnostic tools included selected modules from the Schedules for Clinical Assessment in Neuropsychiatry^19^ and the ICD-10^20^ research criteria for PTSD and depression. Assessment of PTSD was further supported by the PTSD module of the International Trauma Interview in accordance with ICD-11.^21,22^ In addition, baseline outcome measures were collected as outlined below.

**Table 1:** Inclusion and exclusion criteria for the randomised controlled trial Table 1 long description.

Inclusion criteria	Exclusion criteria
Adult (18 years or older) Refugee or family reunified with a refugee PTSD pursuant to the ICD-10 research criteria Psychological trauma experienced outside Denmark Unemployed and assigned to the employment services^a^ in a collaborating municipality Signed informed consent	Severe psychotic disorder (defined as patients with an ICD-10 diagnosis F2x and/or F30.1–F31.9). Participants were excluded only if the psychotic experiences were assessed to be part of an independent psychotic disorder and not part of severe PTSD and/or depression. Dependence syndrome of drugs or alcohol: active dependence and use (F1x.24–F1x.26).

PTSD, post-traumatic stress disorder.

a. This includes patients receiving cash benefits of various types (e.g., social assistance, integration allowance). Disability pension and sickness benefits are not included.

### Treatment as usual

The TAU programme was developed and culturally adapted for refugees and migrants with PTSD receiving care at the CTP and has been refined over more than two decades of clinical practice and research. TAU consisted of 8–12 months of multidisciplinary treatment, including ten sessions with a physician, 16–21 sessions with a psychologist and one session with a social worker. All sessions were planned to last 45 min. Treatment was divided into two phases: phase 1 consisted of six weekly physician sessions and one initial session with a social worker, whereas phase 2 included four monthly physician sessions and weekly sessions with a psychologist. The physicians included both senior consultants in psychiatry and psychiatry residents, reflecting a range of experience levels. Similarly, the psychologists varied in experience but were all licensed clinical psychologists who received regular supervision. Treatment followed standardised manuals to ensure consistency and involved pharmacological treatment according to an algorithm, psychoeducation and flexible cognitive–behavioural therapy. The treatment programme aimed primarily to reduce PTSD and related mental health symptoms and to improve psychological functioning. The programme reflected routine clinical practice at the CTP, a specialised service for trauma-affected refugees and migrants, but was not representative of all PTSD treatment services in Denmark. Additional social worker sessions were provided individually, typically in response to acute issues (e.g. housing, legal status), but TAU did not include a systematic approach to addressing post-migration stressors, and intersectoral collaboration was limited.

### Integrated care add-on

The integrated care intervention was developed through an iterative co-design process involving clinicians, researchers and municipal employment services. Development included a series of co-design workshops across sectors and a pilot study with five patients, clinicians and employment counsellors. Interview feedback regarding feasibility, acceptability and perceived usefulness of the intervention components was used to inform refinements to procedures and patient materials before trial initiation.

The add-on elements were specifically developed for the present study within the specialised transcultural psychiatry service and built on the clinic’s longstanding experience in delivering culturally sensitive mental healthcare to refugee populations. Drawing on existing evidence and recommendations for integrated care and employment interventions, the model emphasised multidisciplinary collaboration, individualised support, addressing both health and social needs, and building on existing programmes to improve scalability and sustainability.^7,15^

The aims of the intervention were to strengthen coordination between mental health and employment services through structured intersectoral collaboration, facilitate changes in social and practical circumstances, and support progress within the employment services system through coordinated problem-solving and clarification of work capacity, social circumstances and rehabilitation needs. This approach reflected the expectation that many participants would face complex health and social barriers to regular employment.^7,15^

The intervention included three structured intersectoral meetings during treatment, bringing together the patient, clinicians from the CTP and the municipal employment counsellor; other stakeholders participated when relevant. Sessions were held either in person or in a hybrid format and were scheduled to last 45–90 min. The three meetings were scheduled early in treatment (within 2 months), midway (after approximately eight psychologist sessions) and at the end of treatment to support transition planning. The Action Plan, a dynamic, patient-centred tool used across sectors to identify and address stressors across seven domains (employment and/or education, finances, family, social life and leisure, accommodation, physical and mental health, and others) was central to the intervention. In addition to improving communication and coordination between sectors, the Action Plan was intended to facilitate concrete actions to reduce barriers and address post-migration stressors affecting participants’ daily lives and recovery. The Action Plan specified shared goals and coordinated actions, guiding both treatment and intersectoral collaboration.

The intersectoral meetings functioned as anchors for ongoing coordination, problem-solving and implementation of agreed actions across sectors. Preparatory sessions with a CTP social worker supported patients in identifying current stressors and treatment barriers that needed to be addressed. Between the intersectoral meetings, the CTP social worker and the employment counsellor maintained *ad hoc* contact. Additional individual sessions with the social worker were offered as needed to address emerging needs.

#### Concomitant care

No restrictions on concomitant care were imposed or systematically recorded.

### Efficacy measures

Efficacy measures were collected pre- and post-treatment and categorised as primary, secondary or exploratory outcomes. Exploratory outcomes included scales not yet validated in this population. Outcomes comprised self-administered rating scales and observer ratings. Questionnaires were available in Danish, English, Arabic and Farsi. For participants who were not fluent in any of these languages or had limited literacy, a professional interpreter assisted with administration. Established validated translations were used where available. The Post-Migration Living Difficulties Checklist (PMLD) was translated following best-practice procedures, including forward and back-translation. An 8 month follow-up, including clinical and employment-related outcomes, was also conducted; this will be reported in a separate paper.

#### Primary outcome

The primary outcome was level of functioning measured using the interviewer-administered 12-item version of the World Health Organization Disability Assessment Schedule 2.0 (WHODAS).^23^ WHODAS was selected as the primary outcome as the aims of the intervention were to improve overall functioning and social participation, rather than symptoms alone. In refugee populations with longstanding PTSD and complex post-migration stressors, functioning represents a clinically meaningful and policy-relevant outcome. WHODAS has demonstrated high internal consistency, test–retest reliability and cross-cultural validity.

#### Secondary outcomes

Self-administered secondary outcomes were scores on the Harvard Trauma Questionnaire (HTQ) (Part IV, 16 items)^24^ for PTSD symptoms, Hopkins Symptom Checklist 25 (HSCL)^25^ for anxiety and depression symptoms, World Health Organization Five Well-Being Index (WHO-5)^26^ for quality of life and Sheehan Disability Scale (SDS)^27^ for level of functioning. Observer ratings used the 17-item Hamilton Rating Scale for Depression (HRSD),^28^ Hamilton Rating Scale for Anxiety (HRSA)^29^ and Global Assessment of Functioning – Symptoms and Functioning (GAF-S and GAF-F).^30^

#### Exploratory outcomes

Self-administered explorative outcomes were scores on the PMLD^31^ for level of post-migration stressors, European Quality of Life 5 Dimensions 5 Levels^32^ for quality of life and Consumer Health Activation Index^33^ for patient activation and empowerment.

#### Process measures

The number and content of treatment sessions were recorded, as well as any instances of dropout. At the end of treatment, patients completed a questionnaire on treatment satisfaction. The questionnaire consisted of five items, rated on a scale of 1 to 9, and was developed at the CTP.^34^

#### Completer criteria and blinding

Treatment completion was defined as having attended at least five physician sessions, at least 10 psychologist sessions and, for the intervention group, at least two intersectoral meetings. Given the nature of the intervention, blinding of participants and clinicians was not feasible. Pre-treatment WHODAS, GAF-F and GAF-S assessments were performed before randomisation and thus blinded to group allocation, but post-treatment assessments were not blinded. The observer-rated measures (HRSD and HRSA) were conducted blinded at both time points. All statistical analyses were performed blinded to group allocation, and the final conclusions were also written blinded.

#### Harms

Adverse events were defined as any serious mental health event or suicide attempt. Health service contacts (e.g. hospital visits) were systematically monitored via trial software alerts and screened by the study team.

### Statistical analyses

#### Power calculation and sample size

At the time of trial design, no established minimal clinically important difference existed for the primary outcome, WHODAS. On the basis of clinical experience and the limited literature, we conservatively estimated an minimal clinically important difference of 5 points and a within-group standard deviation of 10, requiring 64 participants per group (total *n* = 128) for 80% power at *α* = 0.05. On the basis of previous RCTs at the CTP, we accounted for an expected 35% dropout rate and increased the sample size to 197. For secondary outcomes, with 80% power and Cohen’s *d* = 0.5, detectable changes included 0.21 (HTQ), 3.03 (HRSD), 3.77 (HRSA), 0.25 (HSCL), 3.0 (SDS), 8.0 (WHO-5), 4.05 (GAF-F) and 2.85 (GAF-S).

#### Data analyses

The primary analysis compared changes in WHODAS functioning scores from pre- to post-treatment between the intervention and TAU groups, using analysis of covariance (ANCOVA) and linear regression adjusted for baseline scores. Missing data were addressed using multiple imputations. Analyses followed the intention-to-treat (ITT) principle. Secondary outcomes were analysed similarly using ANCOVA and linear regression. Sensitivity analyses were conducted to assess the robustness of the primary results and to supplement the main findings. Baseline covariates that showed imbalances between groups were included in the adjusted outcome models. Differences between participants with and without missing post-treatment data were assessed on the basis of baseline characteristics. To evaluate the impact of missing data, outcomes were also calculated using imputed values equal to the mean ± 2 s.d. of the outcome variable. To assess the influence of extreme values, an additional sensitivity analysis excluding observations beyond ±2 s.d. of the outcome distribution was conducted. Observed case and per-protocol analyses were carried out, and per-protocol results were compared with ITT results to assess the effects of non-adherence. Given that the COVID-19 pandemic overlapped with a substantial portion of the study period, *post hoc* subsample analyses were conducted to compare participants enrolled during 2020–2021 with those enrolled during 2022–2024. Statistical analyses were conducted using Stata Statistical Software, Release 18 (StataCorp LLC, College Station, Texas, USA; https://www.stata.com).

## Results

**Fig. 1:**
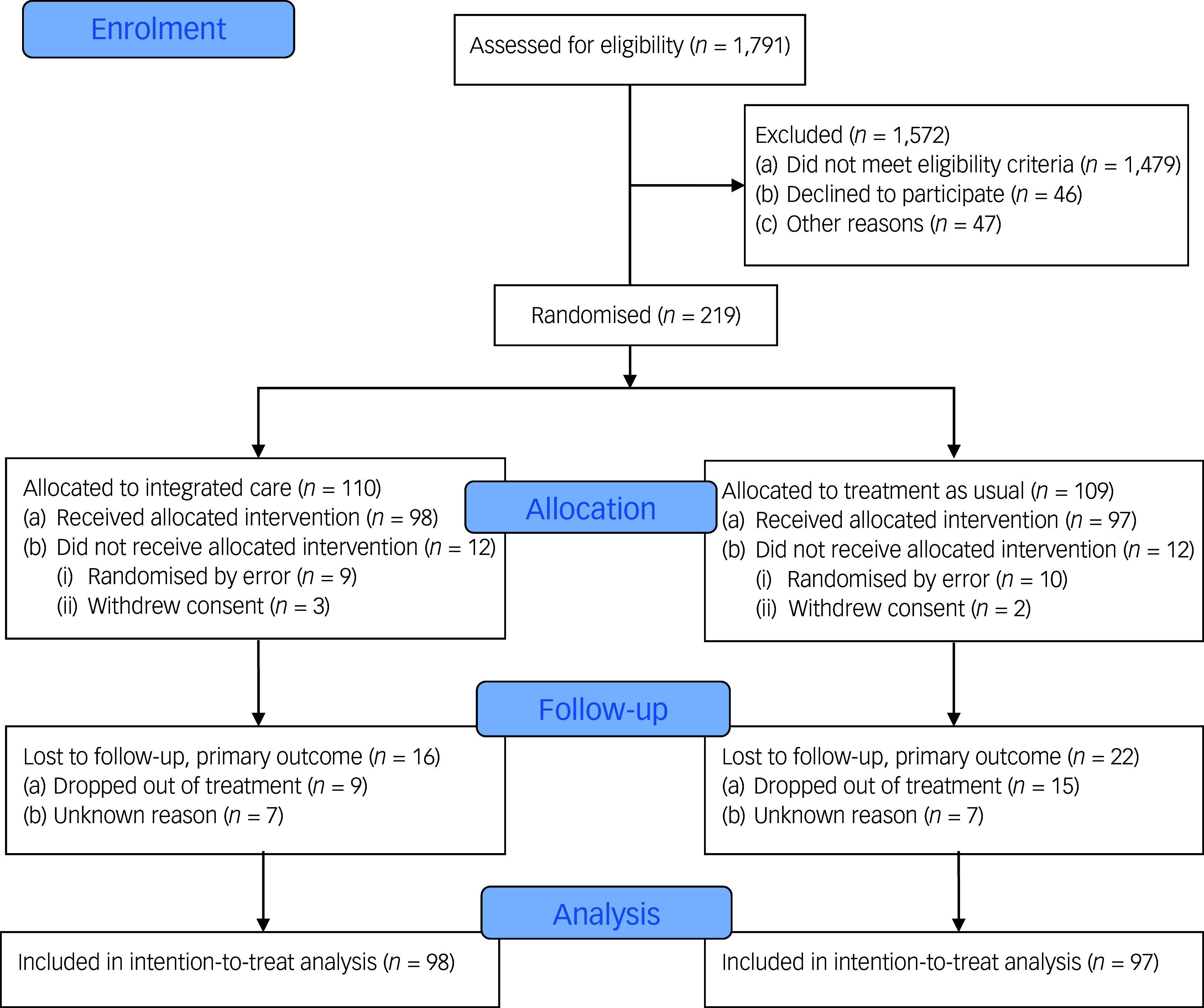
CONSORT flow diagram.

Participant inclusion took place from February 2020 to June 2024. Of the 2058 patients referred to treatment, 1791 attended an initial assessment and were screened for eligibility. Approximately half of these patients resided outside the five trial municipalities, and one-quarter did not have refugee status or were not family reunified with a refugee. Thus, the majority of exclusions reflected the organisational design of the trial, which required residence in a participating municipality and refugee status. After application of all eligibility criteria, 1479 patients were found to be ineligible, 46 declined to participate and 47 were not included for other reasons, primarily because patients decided not to initiate treatment or opted for alternative treatment pathways outside the standard programme of physician and psychologist sessions. A total of 24 participants, equally distributed across groups, were excluded following randomisation owing to eligibility assessment errors (most commonly related to receiving sick leave benefits; *n* = 19) or withdrawal of consent (*n* = 5). These eligibility errors were identified after randomisation. To ensure the planned sample size was achieved, recruitment and randomisation were continued until the target number of eligible participants was reached, resulting in 219 randomised participants. The modified ITT sample thus included 195 participants. The final patient completed the trial in April 2025. See Fig. 1 for the CONSORT flow diagram.

### Participant characteristics

The mean age of the study sample was 47.1 years (s.d. 9.1), and 63.1% of participants were female. Full baseline characteristics across intervention groups are presented in Table 2. The only imbalanced variable at baseline was the mean duration of psychiatric symptoms: 20.4 years (s.d. 13.7) in the integrated care group versus 15.3 years (s.d. 11.9) in the TAU group. See Table 2 for full participant characteristics.

**Table 2:** Sociodemographic characteristics of the study sample Table 2 long description.

Sociodemographic characteristics	All (*n* = 195)	Integrated care (*n* = 98)	Treatment as usual (*n* = 97)	*P*-value
	Mean (s.d.)			
Mean age (*n* = 195)	47.1 (9.1)	47.7 (9.1)	46.5 (9.2)	0.373
Years since arrival in Denmark (*n* = 193)	19.8 (11.3)	20.0 (11.2)	19.6 (11.4)	0.798
Years of work in Denmark (*n* = 191)	4.7 (6.8)	5.2 (7.1)	4.1 (6.6)	0.278
Years of psychiatric symptoms (*n* = 187)	17.9 (13.0)	20.4 (13.7)	15.3 (11.9)	0.007
Years of functional impairment (*n* = 188)	9.1 (8.9)	9.5 (9.3)	8.6 (8.5)	0.519
	*n* (%)			
Gender (*n* = 195)				0.084
Female	123 (63.1)	56 (57.1)	67 (69.1)	
Male	72 (36.9)	42 (42.9)	30 (30.9)	
Country of origin (*n* = 195)				0.345
Iraq	42 (21.5)	20 (20.4)	22 (22.7)	
Lebanon	32 (16.4)	17 (17.4)	15 (15.5)	
Somalia	25 (12.8)	11 (11.2)	14 (14.4)	
Iran	21 (10.8)	6 (6.1)	15 (15.5)	
Afghanistan	18 (9.2)	11 (11.2)	7 (7.2)	
Syria	18 (9.2)	9 (9.2)	9 (9.3)	
Other	39 (20.0)	24 (24.5)	15 (15.5)	
Migrant status (*n* = 195)				0.478
Refugee	130 (66.7)	63 (64.3)	67 (69.1)	
Family reunified with a refugee	65 (33.3)	35 (35.7)	30 (30.9)	
Residence permit (*n* = 189)				0.420
Temporary	70 (37.0)	31 (32.6)	39 (41.5)	
Permanent	54 (28,6)	28 (29.5)	26 (27.7)	
Danish citizenship	65 (34.4)	36 (37.9)	29 (30.9)	
Civil status: having a partner (*n* = 192)	59 (30.7)	31 (31.6)	28 (29.8)	0.782
Having minor children (*n* = 191)	96 (50.2)	48 (49.5)	48 (51.1)	0.827
Highest level of education (*n* = 190)				0.691
None or lower than primary school	59 (31.1)	28 (28.9)	31 (33.3)	
Primary school	50 (26.3)	29 (29.9)	21 (22.6)	
High school	48 (25.3)	23 (23.7)	25 (26.9)	
Higher education	33 (17.4)	17 (17.5)	16 (17.2)	
Need of interpreter (*n* = 195)	122 (62,6)	63 (64.3)	59 (60.8)	0.618
Trauma				
War (*n* = 191)	186 (97.4)	94 (97.9)	92 (96.8)	0.642
Imprisonment (*n* = 191)	46 (24.1)	25 (26.0)	21 (22.1)	0.525
Torture (*n* = 188)	42 (22.3)	25 (26.3)	17 (18.3)	0.186
Domestic violence (*n* = 183)	81 (44.3)	43 (45.3)	28 (43.2)	0.777
Cranial trauma (*n* = 167)	80 (47.9)	40 (46.5)	40 (49.4)	0.710
Treatment and mental health				
Previous psychotherapy (*n* = 161)	69 (42.9)	37 (44.6)	32 (41.0)	0.649
Current and/or previous psychotropics (*n* = 195)	119 (61.0)	60 (61.2)	59 (60.8)	0.954
Current comorbid depression (*n* = 189)	164 (86.8)	82 (87.2)	82 (86.3)	0.852

TAU, treatment as usual.

### Treatment

The mean treatment duration was 10.8 months (s.d. 4.1). Participants received a mean of 9.8 physician sessions (s.d. 3.3). Significant group differences were observed for numbers of psychologist sessions (*P* = 0.018; integrated care: mean 13.5, s.d. 5.7; TAU: mean 11.5, s.d. 5.7), social worker sessions (*P* < 0.001; integrated care: mean 4.4, s.d. 1.7; TAU: mean 2.0, s.d. 1.4) and intersectoral meetings (*P* < 0.001; integrated care: mean 2.4, s.d. 0.8; TAU: mean 0.0, s.d. 0.1). See Supplementary Table 1 available at https://doi.org/10.1192/bjo.2026.12070. No serious adverse events related to the intervention were identified.

### Dropout

In total, 149 participants met completion criteria. Dropout was distributed as follows: one patient before the first treatment session, 13 patients in phase 1 (1–6 physician sessions, no psychologist sessions) and 32 patients during phase 2 (≥6 physician sessions, 1–9 psychologist sessions). In the integrated care group, significantly fewer participants dropped out in phase 1 (*P* = 0.042; integrated care: *n* = 3, 3.1%; TAU: *n* = 10, 10.3%), but no significant differences were observed in phase 2 or with respect to numbers of completers (Supplementary Table 1). Age was the only imbalanced variable between groups for both dropout and missing post-treatment data for the primary outcome, WHODAS, with younger age favouring non-completion as well as missing WHODAS post-treatment score (Supplementary Tables 2 and 3).

### Fidelity of integrated care intervention

All patients in the integrated care group had an initial meeting with the CTP social worker at the start of treatment. On average, participants had one additional social worker session during treatment (excluding sessions linked to intersectoral meetings).

The initial intersectoral meeting (in phase 1) was held for 95.9% of participants (77.4% in person, 22.6% virtual), with 96.8% receiving a preparatory session with the CTP social worker. The Action Plan was used in 97.9% of meetings. The midway intersectoral meeting (in phase 2) was held for 81.6% of participants (60.0% in person, 40.0% virtual), with 79.5% receiving a preparatory session with the CTP social worker. The Action Plan was used in all meetings. The final intersectoral meeting (end of treatment) was held for 66.3% of participants (67.2% in person, 32.8% virtual), with 68.3% receiving a preparatory session. The Action Plan was used in 95.4% of these meetings. The CTP social workers assessed that 92.4% of participants in the integrated care group received an enhanced social intervention compared with TAU.

### Outcomes

We found no significant differences in the primary outcome, WHODAS score, between the integrated care and TAU groups in the ITT analysis (mean difference: 0.30; 95% CI −2.40 to 3.00; *P* = 0.825; integrated care: mean 0.11; TAU: mean 0.41). Similarly, no significant between-group differences were observed for any secondary or exploratory outcomes. Changes in outcome from pre- to post-treatment across measures were generally limited. Full results of the main outcome analyses are presented in Table 3.

**Table 3:** Group differences in outcomes (*n* = 195); intention-to-treat sample with multiple imputation and adjustment for baseline scores using ANCOVA/linear regression Table 3 long description.

Outcome	Add-on integrated care			TAU			Group difference^a^	95% CI	*P*-value
	Baseline score	End score	Diff. score	Baseline score	End score	Diff. score			
WHODAS	30.39	30.38	0.11	29.41	29.95	0.41	0.30	−2.40 to 3.00	0.825
HRSD	23.31	23.59	0.25	23.47	23.89	0.44	0.19	−2.12 to 2.51)	0.869
HRSA	33.29	34.56	1.31	33.07	35.07	1.96	0.65	−3.06 to 4.36)	0.730
GAF-F	47.74	50.16	2.45	47.60	50.31	2.69	0.24	−2.25 to 2.73	0.850
GAF-S	49.47	52.05	2.75	48.62	51.72	2.93	0.18	−2.35 to 2.72	0.886
HTQ	3.09	2.93	−0.16	3.12	2.93	−0.19	−0.02	−0.19 to 0.14	0.777
HSCL	2.97	2.76	−0.21	2.97	2.90	−0.07	0.14	−0.04 to 0.32	0.124
WHO-5	18.67	24.54	6.84	14.78	20.92	5.16	−1.69	−8.54 to 5.16	0.627
SDS	22.98	21.70	−1.06	22.21	21.30	−1.14	−0.08	−2.29 to 2.12	0.941
PMLD	7.25	7.50	−0.81	6.48	7.19	−0.27	0.54	−0.58 to 1.66	0.339
CHAI	58.86	59.23	0.72	56.80	58.03	0.88	0.16	−5.22 to 5.54	0.952
EQ-5D-5L	0.07	0.12	0.04	0.10	0.05	−0.04	−0.08	−0.20 to 0.04	0.166

ANCOVA, analysis of covariance; TAU, treatment as usual; diff., estimated change from baseline to end of treatment; WHODAS, World Health Organization Disability Assessment Schedule 2.0 short form; HRSD, Hamilton Rating Scale for Depression 17; HRSA, Hamilton Rating Scale for Anxiety; GAF-F, Global Assessment of Functioning – Function; GAF-S, Global Assessment of Functioning – Symptoms; HTQ, Harvard Trauma Questionnaire; HSCL, Hopkins Symptom Checklist 25; WHO-5, World Health Organization-Five Well-Being Index; SDS, Sheehan Disability Scale; PMLD, Post-Migration Living Difficulties Checklist; CHAI, Consumer Health Activation Index; EQ-5D-5L, European Quality of Life 5 Dimensions 5 Levels.

a. Group differences were adjusted for baseline score using ANCOVA/linear regression.

### Sensitivity analyses

Results were robust across per-protocol and observed-case analyses (Supplementary Tables 4–10). However, in ITT models adjusted for pre-treatment imbalance in duration of psychiatric symptoms, the integrated care group showed a significantly greater symptom reduction in HSCL compared with TAU (*P* = 0.025). This finding for HSCL was also observed in the sensitivity analysis excluding outliers (±2 s.d.) (*P* = 0.015). Supplementary Table 11 presents the results of statistical assumption tests. For the *post hoc* COVID-19 subsample analysis, no statistically significant group differences were observed (Supplementary Tables 11–14). Missing values on outcomes are reported in Supplementary Table 14.

### Treatment satisfaction

In the treatment satisfaction questionnaire, the two intervention-specific items were significantly higher in the integrated care group compared with TAU: satisfaction with ‘coordination between treatment and employment services*’* (mean difference: 1.14; 95% CI 0.20 to 2.08; *P* = 0.018; integrated care: mean 7.94, s.d.1.91; TAU: mean 6.80, s.d. 3.28) and ‘overview of health and social situation’ (mean difference: 1.05; 95% CI 0.26 to 1.84; *P* = 0.010; integrated care: mean 7.85, s.d. 1.75; TAU: mean 6.80, s.d. 2.64). Owing to a translation error, all Arabic responses to the item on overall satisfaction were excluded from the analysis. Despite this, overall satisfaction remained significantly higher in the integrated care group (mean difference: 1.05; 95% CI 0.08 to 2.04; *P* = 0.035; integrated care: mean 8.20, s.d. 1.68; TAU: mean 7.14, s.d. 2.56). Treatment satisfaction results are shown in Table 4.

**Table 4:** *t*-test of group differences in patient satisfaction post-treatment Table 4 long description.

Aspect of satisfaction^a^	Add-on integrated care		Treatment as usual		Difference	95% CI	*P*-value
	Mean	s.d.	Mean	s.d.			
Intersectoral coordination (*n* = 122)	7.94	1.91	6.80	3.28	1.14	0.20 to 2.08	0.018
Overview of health and social situation (*n* = 122)	7.85	1.75	6.80	2.64	1.05	0.26 to 1.84	0.010
Included what is important to you (*n* = 122)	7.82	1.85	7.44	2.23	0.38	−0.35 to 1.12	0.300
Understanding of cultural background (*n* = 122)	8.06	1.34	7.73	2.27	0.33	−0.32 to 0.99	0.317
Overall satisfaction (*n* = 76^b^)	8.20	1.68	7.14	2.56	1.05	0.08 to 2.03	0.035

a. Scale range: 0–9.

b. Arabic version excluded (owing to an error in translation).

## Discussion

This is the first RCT to investigate an integrated care intervention for refugees with PTSD. The study demonstrated high feasibility of the intervention in a real-world mental health setting. We found no significant differences in clinical outcomes between the intervention groups, and overall change from pre- to post-treatment was limited. However, process measures showed significantly lower levels of early dropout and higher levels of treatment satisfaction in the add-on integrated care group compared with TAU.

To aid interpretation of the trial findings, we drew on insights from a separate qualitative study of 24 patients in the integrated care group; these data were not part of the present trial and are reported elsewhere.^35–37^

### Interpretation and mechanisms

The limited overall change observed across both treatment groups is noteworthy. Although participants received specialised multidisciplinary treatment, improvements were generally modest. The characteristics of the study population may help to explain these findings. Participants showed indicators of chronicity: longstanding symptoms and functional impairment, and extensive previous treatment attempts with psychotherapy and pharmacotherapy. They also had low educational levels, a prolonged residence in Denmark and limited work experience. A 2025 systematic review identified disability, longer time since arrival and pain as negative predictors of treatment outcomes, whereas higher education, employment and female gender were positive predictors.^8^ The characteristics of our sample were largely aligned with these negative predictors. Another key observation was the combination of longstanding functional impairment and minimal labour market integration. Participants had on average experienced psychiatric symptoms and lived in Denmark for about 20 years yet had worked for fewer than 5 years. The qualitative findings suggest that most were unlikely to enter regular employment.^37^ Despite this, eligibility assessments for regular employment or disability benefits were still ongoing.^37^ This pattern points to a potential mismatch between longstanding illness and prolonged unemployment on the one hand, and delayed or unresolved clarification within employment services on the other hand, as well as delayed access to specialised mental healthcare. Such gaps in coordination across sectors may have contributed to the persistence of difficulties. An additional consideration when interpreting the findings is the nature of the comparator. TAU was a highly specialised, culturally adapted, multidisciplinary treatment programme that addressed many of the complex clinical and psychosocial needs of refugees with PTSD. Although TAU did not include structured collaboration with municipal employment services, which characterised the integrated care intervention, its comprehensive nature may have reduced the potential to detect incremental benefits of the add-on intervention. At the same time, the modest overall improvements observed across both groups suggest that the comprehensiveness of TAU alone is unlikely to fully explain the absence of between-group differences.

Direct comparisons with similar trials are challenging owing to heterogeneity in study samples and the predominance of trauma-focused psychotherapy trials in refugee populations, rather than intersectoral or multidisciplinary care models.^3,7^ However, compared with the individuals participating in those studies, participants in the present trial seemed to represent a more chronic and socially marginalised population. Taken together, these findings suggest that the timing of intervention may be critical, and that earlier and more comprehensive mental health and social support during resettlement may be needed to prevent chronic disability and support labour market participation.^38^ This interpretation is consistent with evidence indicating that earlier interventions, before symptoms and functional impairments become entrenched, may be more effective. A low-intensity transdiagnostic intervention, Problem Management Plus (PM+), has shown promising results in reducing psychological symptoms and post-migration stressors in non-clinical refugee samples.^39^

The process evaluation provided insight into potential mechanisms of the intervention. Although no differences were observed in clinical outcomes, the lower rate of early dropout and higher level of treatment satisfaction suggest that the intervention may have enhanced engagement and perceived relevance of care. These findings are consistent with results from integrated care studies in other contexts.^14,40^ Coordinating interventions and addressing post-migration stressors probably helped to stabilise daily life and support attendance. Findings from the accompanying qualitative study indicate that a broad range of post-migration stressors were actively addressed in practice; this included efforts to alleviate challenges related to housing conditions, financial strain, somatic illness, social relations and family support.^35^ A key enabling factor was professionals’ willingness to take on tasks beyond their main areas of responsibility to adopt a needs-based approach to care.^35^ Intersectoral meetings also seemed to function as a pathway to person-centred care, as such collaborations fostered trust, improved communication and promoted a more holistic understanding of patients’ needs.^36^ In addition, collaboration with employment case counsellors seemed to foster a renewed sense of hope and motivation, as patients experienced progress in resolving practical challenges and in advancing their employment clarification process.^36,37^ Higher satisfaction may reflect not only the perceived benefits of additional support but also a more person-centred experience of care. Enhanced engagement and perceived relevance of care may represent important preconditions for effective treatment. However, the add-on components may have been too limited in intensity or duration to produce measurable improvements in clinical outcomes. This interpretation is further supported by the absence of between-group differences in post-migration stressors, one of the key targets of the intervention. The intervention was designed to address practical and social barriers through intersectoral collaboration, and reductions in post-migration stressors were expected to support improvements in mental health and functioning. However, no significant differences were observed on the PMLD measure, suggesting that the intervention did not produce measurable changes in this hypothesised pathway. Taken together, these findings suggest that the intervention may primarily have influenced coordination, patient engagement and perceived relevance of care, without producing measurable improvements in post-migration stressors, symptoms or functioning during the treatment period.

The choice of outcome measures is also relevant when interpreting the findings. The intervention was designed not only to improve coordination between sectors but also to reduce post-migration stressors and practical barriers affecting daily functioning. This rationale was informed by evidence indicating that post-migration stressors may contribute to both psychological distress and functional impairment among refugees. WHODAS score was selected as the primary outcome because functioning was considered to be a clinically meaningful end-point reflecting both mental health and social participation. However, some WHODAS domains may be less sensitive to short-term change, particularly in a population with longstanding symptoms, disability and complex social problems. Other potentially relevant outcomes, including progress in employment clarification processes, social participation and patient-defined recovery goals, were not assessed in the present study. The timing of assessment may also be relevant when interpreting the findings. As part of a broader mixed-methods research programme, which includes a qualitative study and a planned health economic evaluation, this trial contributes to a more comprehensive assessment of the intervention. Several outcomes, particularly those related to employment and engagement with services, are likely to emerge over a longer time frame. The qualitative findings indicate that clarification processes within the employment services often occurred more than 1 year after treatment completion.^37^ A follow-up study is therefore planned to examine longer-term clinical outcomes, employment status and social recovery.

### Generalisability

Approximately half of the participants were included during the COVID-19 pandemic (2020 to early 2022). Pandemic-related restrictions disrupted face-to-face sessions, potentially affecting TAU components and, in particular, integrated care components. A longitudinal study of resettled refugees in Australia found that pandemic-related uncertainty linked to visa status, family separation and the broader COVID-19 context predicted significant increases in symptoms and functional disability.^41^ Moreover, a study found that increased rates of mental illness among refugees persisted for 4 years post-COVID-19.^42^ Lockdowns may also have exacerbated many post-migration stressors and basic needs, whereas demands from employment services may have decreased during lockdowns, potentially alleviating some stressors related to employment services obligations. Together, these factors may have influenced both intervention delivery and outcomes and should be considered when interpreting the generalisability of the findings to non-pandemic conditions.

The generalisability of the findings should also be interpreted in light of the study sample and setting. Although many screened participants did not meet eligibility criteria (primarily regarding municipality of residence and refugee status), the study population is likely to have been representative of the intended target population of unemployed refugees with PTSD receiving specialised mental health treatment. Given the characteristics of the study sample, the findings may not be generalisable to more recently arrived refugees. Other context-dependent factors should also be considered when interpreting these findings. The integrated care intervention was developed in a Western European context; however, its core components – structured intersectoral meetings, coordination and shared care plans – are not country-specific. A 2024 review of multisectoral humanitarian programmes identified similar principles in low- and middle-income settings.^43^ Adaptation to local policy and funding contexts is needed, but the model is aligned with global moves towards integrated, person-centred and recovery-oriented mental health systems.^13,16^

### Strengths and limitations

Strengths of this study include its large, real-world RCT design and comprehensive assessment of clinical outcomes and process measures. The integrated care intervention utilised low-cost, feasible and scalable components. The number of completers exceeded the power-calculated sample size, strengthening the robustness of the analyses.

We acknowledge several limitations of the study. First, outcome assessors were not blinded post-treatment for all measures, which may have introduced assessment bias. WHODAS and GAF assessments were conducted at baseline before randomisation and were therefore blinded by group; however, post-treatment assessments were not blinded, reflecting the pragmatic design of the trial within routine clinical practice, where blinded assessment of these measures was not feasible. By contrast, the observer-rated measures HRSD and HRSA were assessed under blinded conditions at both time points. Second, although multiple imputation was applied, missing data may have affected estimates. Third, the pragmatic design introduced clinical variability, as treatment length, psychotherapeutic modalities and medication use varied according to individual needs. Although this enhanced the real-world relevance of the study, it reduced experimental control. In addition, variability in the implementation of the intersectoral components, including differences in local employment service resources and the quality of intersectoral collaboration, may have contributed to heterogeneity in delivery of the add-on intervention. Finally, the use of TAU as the comparator may have limited our ability to detect incremental effects of the add-on integrated care intervention, although overall improvements in both groups were modest.

### Clinical implications

Many refugees receiving mental healthcare experience longstanding symptoms, functional impairment and complex social challenges. The findings of this study suggest that add-on integrated care is unlikely to produce measurable clinical or functional improvements when delivered late in the course of illness. This highlights the importance of realistic expectations regarding the impact of short-term, add-on interventions in populations with chronic and complex needs. From a service perspective, structured intersectoral collaborations such as shared care plans and joint meetings may still support coordination, continuity of care and patient engagement

### Recommendations for future research

Given the limited evidence base for integrated care in refugee populations, future research should aim to identify which components of integrated care are most effective, and for whom. Studies should examine whether integrated care is more effective when implemented earlier in the resettlement process, before post-migration stressors and functional impairments become entrenched. Further research is also needed to determine how integrated care can be adapted for refugees with longstanding and complex needs, including provision of the intensity, duration and continuity of support required to achieve meaningful improvements. Mixed-methods and person-centred approaches may help to capture heterogeneity in response and clarify mechanisms of action. As many potential benefits of integrated care, such as labour market participation, social integration and healthcare utilisation, are unlikely to emerge within a single treatment episode, future studies should include longer-term follow-up and broader outcome measures.

## Supporting information

10.1192/bjo.2026.12070.sm001 Bruhn et al. supplementary material Bruhn et al. supplementary material

## Data Availability

The protocol has been published. The research materials (protocols, treatment manuals, consent forms, etc.) will be available from the corresponding author upon request. The data are not publicly available owing to information that could compromise the privacy of research participants. The statistical code and aggregated data supporting the findings of this study are available from the corresponding author upon reasonable request, subject to the limits of Danish Data Protection legislation.

## Supplementary material

The supplementary material is available online at https://doi.org/10.1192/bjo.2026.12070

## Table 1 Long description

A table with two columns and six rows. The columns are labeled Inclusion criteria and Exclusion criteria. The rows list the specific criteria under each column. Row 1: Inclusion criteria: Adult 18 years or older, Exclusion criteria: Severe psychotic disorder defined as patients with an ICD-10 diagnosis F2x and/or F30.1-F31.9. Participants were excluded only if the psychotic experiences were assessed to be part of an independent psychotic disorder and not part of severe PTSD and/or depression. Row 2: Inclusion criteria: Refugee or family reunited with a refugee, Exclusion criteria: Dependence syndrome of drugs or alcohol: active dependence and use F1x.24-F1x.26. Row 3: Inclusion criteria: PTSD pursuant to the ICD-10 research criteria, Exclusion criteria: None. Row 4: Inclusion criteria: Psychological trauma experienced outside Denmark, Exclusion criteria: None. Row 5: Inclusion criteria: Unemployed and assigned to the employment services in a collaborating municipality, Exclusion criteria: None. Row 6: Inclusion criteria: Signed informed consent, Exclusion criteria: None.

Navigate back to Table 1.

## Table 2 Long description

A table comparing sociodemographic characteristics of a study sample across integrated care and treatment as usual groups. The table has 19 rows and 6 columns. Column headers are Sociodemographic characteristics, All (n = 195), Integrated care (n = 98), Treatment as usual (n = 97), and P-value. Row labels include Mean age (s.d.), Years since arrival in Denmark (n = 193), Years of work in Denmark (n = 191), Years of psychiatric symptoms (n = 187), Years of functional impairment (n = 188), Sex (n = 195), Country of origin (n = 195), Migrant status (n = 195), Residence permit (n = 189), Civil status: having a partner (n = 192), Having minor children (n = 191), Highest level of education (n = 190), Need of interpreter (n = 195), War (n = 191), Imprisonment (n = 191), Torture (n = 188), Domestic violence (n = 183), Cranial trauma (n = 167), Previous psychotherapy (n = 161), Current and/or previous psychotropics (n = 195), and Current comorbid depression (n = 189). Each row provides specific values for each column, detailing the sociodemographic characteristics and their distribution across the two groups.

Navigate back to Table 2.

## Table 3 Long description

The table compares scores of various outcomes between two care groups: Add-on integrated care and TAU. It includes baseline scores, end scores, and difference scores for each outcome. The outcomes listed are WHODAS, HRSD, HRSA, GAF-F, GAF-S, HTQ, HSCL, WHO-5, SDS, PMLD, CHAI, and EQ-5D-5L. The table also shows group differences, 95% confidence intervals, and P-values for each outcome. Row 1: WHODAS, Add-on integrated care Baseline score 30.39, End score 30.38, Diff. score 0.11, TAU Baseline score 29.41, End score 29.95, Diff. score 0.41, Group difference 0.30, 95% CI -2.40 to 3.00, P-value 0.825. Row 2: HRSD, Add-on integrated care Baseline score 23.31, End score 23.59, Diff. score 0.25, TAU Baseline score 23.47, End score 23.89, Diff. score 0.44, Group difference 0.19, 95% CI -2.12 to 2.51, P-value 0.869. Row 3: HRSA, Add-on integrated care Baseline score 33.29, End score 34.56, Diff. score 1.31, TAU Baseline score 33.07, End score 35.07, Diff. score 1.96, Group difference -0.65, 95% CI -3.06 to 4.36, P-value 0.730. Row 4: GAF-F, Add-on integrated care Baseline score 47.74, End score 50.16, Diff. score 2.45, TAU Baseline score 47.60, End score 50.31, Diff. score 2.69, Group difference 0.24, 95% CI -2.25 to 2.73, P-value 0.850. Row 5: GAF-S, Add-on integrated care Baseline score 49.47, End score 52.05, Diff. score 2.75, TAU Baseline score 48.62, End score 51.72, Diff. score 2.93, Group difference 0.18, 95% CI -2.35 to 2.72, P-value 0.886. Row 6: HTQ, Add-on integrated care Baseline score 3.09, End score 2.93, Diff. score -0.16, TAU Baseline score 3.12, End score 2.93, Diff. score -0.19, Group difference 0.02, 95% CI -0.19 to 0.14, P-value 0.777. Row 7: HSCL, Add-on integrated care Baseline score 2.97, End score 2.76, Diff. score -0.21, TAU Baseline score 2.97, End score 2.90, Diff. score -0.07, Group difference -0.14, 95% CI -0.40 to 0.32, P-value 0.124. Row 8: WHO-5, Add-on integrated care Baseline score 18.67, End score 24.54, Diff. score 6.84, TAU Baseline score 14.78, End score 20.92, Diff. score 5.16, Group difference -1.69, 95% CI -8.54 to 5.16, P-value 0.627. Row 9: SDS, Add-on integrated care Baseline score 22.98, End score 21.70, Diff. score -1.06, TAU Baseline score 22.21, End score 21.30, Diff. score -1.14, Group difference 0.08, 95% CI -2.29 to 2.12, P-value 0.941. Row 10: PMLD, Add-on integrated care Baseline score 7.25, End score 7.50, Diff. score 0.81, TAU Baseline score 6.48, End score 7.19, Diff. score -0.27, Group difference 0.54, 95% CI -0.58 to 1.66, P-value 0.339. Row 11: CHAI, Add-on integrated care Baseline score 58.86, End score 59.23, Diff. score 0.72, TAU Baseline score 56.80, End score 58.03, Diff. score 0.88, Group difference 0.16, 95% CI -5.22 to 5.54, P-value 0.952. Row 12: EQ-5D-5L, Add-on integrated care Baseline score 0.07, End score 0.12, Diff. score 0.04, TAU Baseline score 0.10, End score 0.05, Diff. score -0.04, Group difference -0.08, 95% CI -0.20 to 0.04, P-value 0.166.

Navigate back to Table 3.

## Table 4 Long description

The table compares patient satisfaction across various aspects between add-on integrated care and treatment as usual. It has six rows and seven columns. The columns are labeled Aspect of satisfaction, Add-on integrated care Mean, Add-on integrated care s.d., Treatment as usual Mean, Treatment as usual s.d., Difference, 95% CI, and P-value. The rows are labeled with different aspects of satisfaction: Intersectoral coordination, Overview of health and social situation, Included what is important to you, Understanding of cultural background, and Overall satisfaction. Row 1: Intersectoral coordination, Add-on integrated care Mean 7.94, Add-on integrated care s.d. 1.91, Treatment as usual Mean 6.80, Treatment as usual s.d. 3.28, Difference 1.14, 95% CI 0.20 to 2.08, P-value 0.018. Row 2: Overview of health and social situation, Add-on integrated care Mean 7.85, Add-on integrated care s.d. 1.75, Treatment as usual Mean 6.80, Treatment as usual s.d. 2.64, Difference 1.05, 95% CI 0.26 to 1.84, P-value 0.010. Row 3: Included what is important to you, Add-on integrated care Mean 7.82, Add-on integrated care s.d. 1.85, Treatment as usual Mean 7.44, Treatment as usual s.d. 2.23, Difference 0.38, 95% CI -0.35 to 1.12, P-value 0.300. Row 4: Understanding of cultural background, Add-on integrated care Mean 8.06, Add-on integrated care s.d. 1.34, Treatment as usual Mean 7.73, Treatment as usual s.d. 2.27, Difference 0.33, 95% CI -0.32 to 0.99, P-value 0.317. Row 5: Overall satisfaction, Add-on integrated care Mean 8.20, Add-on integrated care s.d. 1.68, Treatment as usual Mean 7.14, Treatment as usual s.d. 2.56, Difference 1.05, 95% CI 0.08 to 2.04, P-value 0.035.

Navigate back to Table 4.
