# Supplementary material for: Integrated mental health treatment and employment services for refugees with PTSD: randomised controlled trial
Source: BJPsych Open. 2026 Aug 5;12(5):e207. doi: 10.1192/bjo.2026.12070 (PMC13444159; doi:10.1192/bjo.2026.12070)
Supplement: Supplementary file 1 [file S2056472426120705sup001.docx]

**Supplementary material**

**Table of content**

[Table S1. Number of treatment sessions and dropout rates by intervention group.	2](#_Toc225112255)

[Table S2. Dropout analysis: Differences in sociodemographic and pre-treatment outcome variables between completers and non-completers.	3](#_Toc225112256)

[Table S3. Lost to follow-up: Differences in sociodemographic and pre-treatment outcome variables between patients with and without missing WHODAS post-treatment data.	5](#_Toc225112257)

[Table S4. Completer analysis of group differences in outcomes (*n* = 195); ANCOVA/linear regression adjusted for baseline scores with multiple imputation.	6](#_Toc225112258)

[Table S5. Intention-to-treat analysis of group differences in outcomes (*n* = 195); ANCOVA/linear regression with multiple imputation, adjusted for baseline scores and years of psychiatric symptoms.	7](#_Toc225112259)

[Table S6. Observed case analysis of group differences in outcomes; ANCOVA/linear regression adjusted for baseline scores.	7](#_Toc225112260)

[Table S7. Low-value scenario of group differences in outcomes (*n* = 195); intention-to-treat sample with missing outcomes imputed as mean –2 SD, adjusted for baseline scores.	8](#_Toc225112261)

[Table S8. High-value scenario of group differences in outcomes (*n* = 195); intention-to-treat sample with missing outcomes imputed as mean +2 SD, adjusted for baseline scores.	8](#_Toc225112262)

[Table S9: Group differences in outcomes without outliers (*n* = 195); intention-to-treat sample with multiple imputation, excluding values ±2 SD, adjusted for baseline scores.	9](#_Toc225112263)

[Table S10. Summary of results from assumption testing.	9](#_Toc225112264)

[Table S11: Number of patients included per year.	10](#_Toc225112265)

[Table S12. Comparison of outcomes between intervention groups in the subsample included during non-pandemic years (2022–2024); intention-to-treat sample (*n* = 101), ANCOVA/linear regression adjusted for baseline scores with multiple imputation.	10](#_Toc225112266)

[Table S13. Comparison of outcomes in the integrated care group during pandemic (2020–2021) vs non-pandemic years (2022–2024); intention-to-treat sample (*n* = 98), ANCOVA/linear regression adjusted for baseline scores with multiple imputation.	11](#_Toc225112267)

[Table S14. Comparison of outcomes in TAU group during pandemic (2020–2021) vs non-pandemic years (2022–2024); intention-to-treat sample (*n* = 98), ANCOVA/linear regression adjusted for baseline scores with multiple imputation.	11](#_Toc225112268)

[Table S15. Available data for all outcomes at each time point.	12](#_Toc225112269)

### Table S1. Number of treatment sessions, dropout rates, and psychotropics by intervention group.

|  | **All** *n*=195 | | **Integrated care** *n*=98 | | **TAU** *n*=97 | | *p*-value |
| --- | --- | --- | --- | --- | --- | --- | --- |
|  | **Mean (SD)** | | | | | |  |
| Treatment length in months | 10.8 | (4.1) | 11.2 | (3.7) | 10.3 | (4.3) | 0.10 |
| No. of sessions |  |  |  |  |  |  |  |
| Physician | 9.8 | (3.3) | 10.2 | (3.2) | 9.4 | (3.4) | 0.12 |
| Psychologist | 12.5 | (6.1) | 13.5 | (5.7) | 11.5 | (6.4) | 0.018 |
| Social worker | 3.2 | (2.0) | 4.4 | (1.7) | 2.0 | (1.4) | <0.001 |
| Intersectoral meetings | 1.2 | (1.3) | 2.4 | (0.8) | 0.0 | (0.1) | <0.001 |
|  | ***n* (%)** | | | | | |  |
| No. of completers | 149 | (76.4) | 79 | (80.6) | 70 | (72.2) | 0.17 |
| Non-completers (dropout) |  |  |  |  |  |  |  |
| Before first treatment session | 1 | (0.5) | 0 | (0.0) | 1 | (1.0) | 0.31 |
| Phase 1 (1-6 physician sessions, no psychologist sessions) | 13 | (6.7) | 3 | (3.1) | 10 | (10.3) | 0.042 |
| Phase 2 (≥6 physician sessions, 1-9 psychologist sessions) | 32 | (16.4) | 16 | (16.3) | 16 | (16.5) | 0.98 |
| Psychotropics at end of treatment |  |  |  |  |  |  |  |
| Antidepressants | 128 | (65.6) | 68 | (69.4) | 60 | (65.6) | 0.28 |
| Antipsychotics | 13 | (6.7) | 5 | (5.1) | 8 | (8.3) | 0.38 |
| Anxiolytics | 5 | (2.6) | 3 | (3.1) | 2 | (2.1) | 0.66 |
| Other | 2 | (1.0) | 2 | (2.0) | 0 | (0.0) | 0.16 |
| ^1^ Completer criteria: ≥5 physician sessions, ≥10 psychologist sessions, and for the integrated care group, ≥2 intersectoral meetings. | | | | | | | |

### Table S2. Dropout analysis: Differences in sociodemographic and pre-treatment outcome variables between completers and non-completers.

|  | **All** | | **Non-completer**  (*n* = 46) | | **Completer**  (*n* = 149) | | ***p*-value** |
| --- | --- | --- | --- | --- | --- | --- | --- |
| **Sociodemographic characteristics** | **Mean (SD)** | | | | | |  |
| Mean age (*n*=195) | 47.1 | (9.1) | 44.3 | (9.6) | 47.9 | (8.8) | 0.019 |
| Years since arrival in Denmark (*n*=193) | 19.8 | (11.3) | 19.0 | (11.5) | 20.1 | (11.2) | 0.59 |
| Years of work in origin (*n*=192) | 4.5 | (7.1) | 3.1 | (5.1) | 4.8 | (7.6) | 0.18 |
| Years of work in Denmark (*n*=191) | 4.7 | (6.8) | 4.5 | (7.5) | 4.7 | (6.7) | 0.84 |
|  | ***n* (%)** | | | | | |  |
| Sex (*n*=195) |  |  |  |  |  |  | 0.73 |
| Female | 123 | (63.1) | 30 | (65.2) | 93 | (62.4) |  |
| Male | 72 | (36.9) | 16 | (34.8) | 56 | (37.6) |  |
| Country of origin (*n*=195) |  |  |  |  |  |  | 0.48 |
| Iraq | 42 | (21.5) | 10 | (21.7) | 32 | (21.5) |  |
| Lebanon | 32 | (16.4) | 7 | (15.2) | 25 | (16.8) |  |
| Somalia | 25 | (12.8) | 4 | (8.7) | 21 | (14.1) |  |
| Iran | 21 | (10.8) | 6 | (13.0) | 15 | (10.8) |  |
| Afghanistan | 18 | (9.2) | 3 | (6.5) | 15 | (10.1) |  |
| Syria | 18 | (9.2) | 7 | (15.2) | 11 | (7.4) |  |
| Ukraine | 13 | (6.7) | 1 | (2.2) | 12 | (8.1) |  |
| Other | 26 | (13.3) | 8 | (17.4) | 18 | (12.1) |  |
| Migrant status (*n*=195) |  |  |  |  |  |  | 0.095 |
| Refugee | 130 | (66.7) | 26 | (56.5) | 104 | (69.8) |  |
| Family reunified with a refugee | 65 | (33.3) | 20 | (43.5) | 45 | (30.2) |  |
| Residence permit (*n*=189) |  |  |  |  |  |  | 0.67 |
| Temporary | 70 | (37.0) | 17 | (40.5) | 53 | (36.1) |  |
| Permanent | 54 | (28,6) | 13 | (31.0) | 41 | (27.9) |  |
| Danish citizenship | 65 | (34.4) | 12 | (28.6) | 53 | (36.1) |  |
| Civil status: Having a partner (*n*=192) | 59 | (30.7) | 10 | (23.3) | 49 | (32.9) | 0.23 |
| Having minor children (*n*=191) | 96 | (50.2) | 20 | (46.5) | 76 | (51.4) | 0.58 |
| Living alone (*n*=190) | 60 | (31.6) | 17 | (39.5) | 43 | (29.3) | 0.20 |
| Highest level of education (*n*=190) |  |  |  |  |  |  | 0.46 |
| No/lower than primary school | 59 | (31.1) | 13 | (30.2) | 46 | (31.3) |  |
| Primary school | 50 | (26.3) | 15 | (34.9) | 35 | (23.8) |  |
| High school | 48 | (25.3) | 8 | (18.6) | 40 | (27.2) |  |
| Higher education | 33 | (17.4) | 7 | (16.3) | 26 | (17.7) |  |
| Need of interpreter (*n*=195) | 122 | (62,6) | 27 | (58.7) | 95 | (63.8) | 0.54 |
| Municipality (*n*=195) |  |  |  |  |  |  | 0.92 |
| Copenhagen | 163 | (83.6) | 40 | (87.0) | 123 | (82.6) |  |
| Gladsaxe | 14 | (7.2) | 2 | (4.4) | 12 | (8.1) |  |
| Hillerød | 8 | (4.1) | 2 | (4.4) | 6 | (4.0) |  |
| Lyngby-Taarbæk | 6 | (3.1) | 1 | (2.2) | 5 | (3.4) |  |
| Frederikssund | 4 | (2.1) | 1 | (2.2) | 3 | (2.0) |  |
| **Trauma** |  |  |  |  |  |  |  |
| War | 186 | (97.4) | 42 | (95.5) | 144 | (97.7) | 0.36 |
| Imprisonment | 46 | (24.1) | 9 | (20.9) | 37 | (25.0) | 0.58 |
| Torture | 42 | (22.3) | 9 | (20.9) | 33 | (22.8) | 0.80 |
| Domestic violence | 81 | (44.3) | 22 | (55.0) | 59 | (41.3) | 0.12 |
| Sexual assaults | 42 | (24.2) | 8 | (22.2) | 34 | (25.0) | 0.73 |
| Cranial trauma | 80 | (47.9) | 16 | (47.1) | 64 | (48.1) | 0.91 |
| **Treatment and mental health** |  |  |  |  |  |  |  |
| Previous psychotherapy (*n*=161) | 69 | (42.9) | 11 | (44.0) | 58 | (42.7) | 0.90 |
| Previous/current psychotropics (*n*=195) | 119 | (61.0) | 26 | (56.2) | 93 | (62.4) | 0.47 |
| Current co-morbid depression (ICD-10) (*n*=189) | 164 | (86.8) | 39 | (88.6) | 125 | (86.2) | 0.68 |
|  |  |  |  |  |  |  |  |
| **Mental health baseline** | **Mean (SD)** | | | | | |  |
| Years of psychiatric symptoms (*n*=187) | 17.9 | (12.9) | 18.7 | (13.7) | 17.7 | (12.9) | 0.68 |
| Years of functional impairment (*n*=188) | 9.1 | (8.4) | 10.5 | (10.4) | 8.6 | (8.4) | 0.23 |
| WHODAS (*n*=187) | 29.9 | (9.0) | 28.7 | (10.8) | 30.3 | (8.4) | 0.32 |
| HDRS (*n*=178) | 23.4 | (5.8) | 24.1 | (5.2) | 23.2 | (5.9) | 0.37 |
| HARS (*n*=178) | 33.2 | (9.1) | 33.9 | (9.5) | 33.0 | (9.0) | 0.58 |
| GAF-S (*n*=195) | 49.0 | (6.3) | 49.4 | (7.3) | 48.9 | (5.7) | 0.65 |
| GAF-F (*n*=195) | 47.7 | (6.9) | 48.2 | (8.8) | 47.5 | (6.2) | 0.54 |
| HTQ (*n*=188) | 3.1 | (0.4) | 3.0 | (0.4) | 3.1 | (0.4) | 0.18 |
| HSCL (*n*=187) | 3.0 | (0.4) | 2.9 | (0.5) | 3.0 | (0.4) | 0.17 |
| WHO-5 (*n*=183) | 16.7 | (15.1) | 18.0 | (19.0) | 16.4 | (13.8) | 0.53 |
| SDS (*n*=179) | 22.5 | (6.1) | 20.8 | (8.3) | 23.0 | (5.3) | 0.046 |
| PMLD (*n*=188) | 7.3 | (3.5) | 7.6 | (3.5) | 7.3 | (3.6) | 0.61 |
| CHAI (*n*=185) | 58.0 | (18.6) | 52.4 | (17.4) | 59.6 | (18.6) | 0.029 |
| EQ-5D-5L (*n*=167) | 0.08 | (0.37) | 0.12 | (0.42) | 0.07 | (0.35) | 0.48 |
| Completer criteria: ≥5 physician sessions, ≥10 psychologist sessions, and for the integrated care group, ≥2 intersectoral meetings.  SD: Standard deviation. WHODAS: The World Health Organization Disability Assessment Schedule 2.0 short form. HDRS: Hamilton Depression Rating Scale 17. HARS: Hamilton Anxiety Rating Scale. GAF-F: Global Assessment of Functioning – Function. GAF-S: Global Assessment of Functioning – Symptoms. HTQ: Harvard Trauma Questionnaire. HSCL: Hopkins Symptom Check List 25. WHO-5: The World Health Organization-Five Well-Being Index. SDS: Sheehan Disability Scale. PMLD: Post-migration Living Difficulties Check List. CHAI: Consumer Health Activation Index. EQ-5D-5L: EuroQol 5-Dimensions 5-Levels. | | | | | | | |

### Table S3. Lost to follow-up: Differences in sociodemographic and pre-treatment outcome variables between patients with and without missing WHODAS post-treatment data.

|  | **All** | | **WHODAS  not missing**  *n* = 157 | | **WHODAS  missing**  *n* = 38 | | ***p*-value** |
| --- | --- | --- | --- | --- | --- | --- | --- |
| **Sociodemographic characteristics** | **Mean (SD)** | | | | | |  |
| Mean age (*n*=195) | 47.1 | (9.1) | 47.8 | (9.1) | 44.0 | (8.8) | 0.021 |
| Years since arrival in Denmark (*n*=193) | 19.8 | (11.3) | 20.5 | (11.1) | 17.2 | (11.7) | 0.11 |
| Years of work in origin (n = 192) | 4.5 | (7.1) | 4.6 | (7.5) | 4.1 | (5.5) | 0.71 |
| Years of work in Denmark (n = 191) | 4.7 | (6.8) | 4.7 | (6.9) | 4.6 | (6.8) | 0.97 |
|  | ***n* (%)** | | | | | |  |
| Sex (*n*=195) |  |  |  |  |  |  | 0.27 |
| Female | 123 | (63.1) | 102 | (65.0) | 21 | (55.3) |  |
| Male | 72 | (36.9) | 55 | (35.0) | 17 | (44.7) |  |
| Country of origin (*n*=195) |  |  |  |  |  |  | 0.16 |
| Iraq | 42 | (21.5) | 33 | (21.0) | 9 | (23.7) |  |
| Lebanon | 32 | (16.4) | 31 | (19.8) | 1 | (2.6) |  |
| Somalia | 25 | (12.8) | 21 | (13.4) | 4 | (10.5) |  |
| Iran | 21 | (10.8) | 17 | (10.8) | 4 | (10.5) |  |
| Afghanistan | 18 | (9.2) | 15 | (9.6) | 3 | (7.9) |  |
| Syria | 18 | (9.2) | 13 | (8.3) | 5 | (13.2) |  |
| Ukraine | 13 | (6.7) | 10 | (6.4) | 3 | (7.9) |  |
| Other | 26 | (13.3) | 17 | (10.8) | 9 | (23.7) |  |
| Migrant status (*n*=195) |  |  |  |  |  |  | 0.90 |
| Refugee | 130 | (66.7) | 105 | (66.9) | 25 | (65.8) |  |
| Family reunified with a refugee | 65 | (33.3) | 52 | (33.1) | 13 | (34.2) |  |
| Residence permit (*n*=189) |  |  |  |  |  |  | 0.20 |
| Temporary | 70 | (37.0) | 53 | (34.4) | 17 | (48.6) |  |
| Permanent | 54 | (28,6) | 44 | (28.6) | 10 | (28.6) |  |
| Danish citizenship | 65 | (34.4) | 57 | (37.0) | 8 | (22.9) |  |
| Civil status: Having a partner (*n*=192) | 59 | (30.7) | 50 | (32.1) | 9 | (25.0) | 0.41 |
| Having minor children (*n*=191) | 96 | (50.2) | 74 | (47.7) | 22 | (61.1) | 0.15 |
| Living alone (*n*=190) | 60 | (31.6) | 48 | (31.2) | 12 | (33.3) | 0.80 |
| Highest level of education (*n*=190) |  |  |  |  |  |  | 0.090 |
| No/lower than primary school | 59 | (31.1) | 51 | (33.1) | 8 | (22.2) |  |
| Primary school | 50 | (26.3) | 35 | (22.7) | 15 | (41.7) |  |
| High school | 48 | (25.3) | 42 | (27.3) | 6 | (16.7) |  |
| Higher education | 33 | (17.4) | 26 | (16.9) | 7 | (19.4) |  |
| Need of interpreter (*n*=195) | 122 | (62,6) | 98 | (62.4) | 24 | (63.2) | 0.93 |
| Municipality (*n*=195) |  |  |  |  |  |  | 0.99 |
| Copenhagen | 163 | (83.6) | 132 | (84.1) | 31 | (81.6) |  |
| Gladsaxe | 14 | (7.2) | 11 | (7.0) | 3 | (7.9) |  |
| Hillerød | 8 | (4.1) | 6 | (3.8) | 2 | (5.3) |  |
| Lyngby-Taarbæk | 6 | (3.1) | 5 | (3.2) | 1 | (2.6) |  |
| Frederikssund | 4 | (2.1) | 3 | (1.9) | 1 | (2.6) |  |
| **Trauma** |  |  |  |  |  |  |  |
| War | 186 | (97.4) | 149 | (96.8) | 37 | (100.0) | 0.27 |
| Imprisonment | 46 | (24.1) | 41 | (26.6) | 5 | (13.5) | 0.094 |
| Torture | 42 | (22.3) | 36 | (23.5) | 6 | (17.1) | 0.41 |
| Domestic violence | 81 | (44.3) | 66 | (44.3) | 15 | (44.1) | 0.99 |
| Sexual assaults | 42 | (24.2) | 35 | (24.7) | 7 | (23.3) | 0.88 |
| Cranial trauma | 80 | (47.9) | 68 | (49.6) | 12 | (40.0) | 0.34 |
| **Treatment and mental health** |  |  |  |  |  |  |  |
| Previous psychotherapy | 69 | (42.9) | 63 | (44.4) | 6 | (31.6) | 0.29 |
| Previous/current psychotropics | 119 | (61.0) | 99 | (63.1) | 20 | (52.6) | 0.24 |
| Current co-morbid depression (ICD-10) | 164 | (86.8) | 132 | (86.3) | 32 | (88.9) | 0.68 |
|  |  |  |  |  |  |  |  |
| **Symptom scores** | **Mean (SD)** | | | | | |  |
| Years of psychiatric symptoms (*n*=187) | 17.9 | (12.9) | 18.3 | (13.1) | 16.4 | (12.8) | 0.45 |
| Years of functional impairment (*n*=188) | 9.1 | (8.4) | 9.0 | (9.0) | 9.6 | (8.3) | 0.72 |
| WHODAS (*n*=187) | 29.9 | (9.0) | 30.3 | (8.6) | 28.2 | (10.5) | 0.21 |
| HDRS (*n*=178) | 23.4 | (5.8) | 23.4 | (6.0) | 23.4 | (4.6) | 0.95 |
| HARS (*n*=178) | 33.2 | (9.1) | 33.3 | (9.1) | 32.7 | (9.4) | 0.78 |
| GAF-S (*n*=195) | 49.0 | (6.3) | 49.1 | (5.8) | 49.0 | (8.0) | 0.94 |
| GAF-F (*n*=195) | 47.7 | (6.9) | 47.4 | (6.6) | 48.6 | (7.8) | 0.35 |
| HTQ (*n*=188) | 3.1 | (0.4) | 3.1 | (0.4) | 3.0 | (0.4) | 0.21 |
| HSCL (*n*=187) | 3.0 | (0.4) | 3.0 | (0.5) | 3.0 | (0.4) | 0.35 |
| WHO-5 (*n*=183) | 16.7 | (15.1) | 17.0 | (15.7) | 15.5 | (11.7) | 0.61 |
| SDS (*n*=179) | 22.5 | (6.1) | 22.7 | (6.0) | 21.6 | (6.8) | 0.39 |
| PMLD (*n*=188) | 7.3 | (3.5) | 7.1 | (3.6) | 8.2 | (3.3) | 0.10 |
| CHAI (*n*=185) | 58.0 | (18.6) | 58.4 | (19.1) | 56.2 | (16.1) | 0.53 |
| EQ-5D-5L (*n*=167) | 0.08 | (0.37) | 0.07 | (0.36) | 0.14 | (0.43) | 0.42 |
| SD: Standard deviation. WHODAS: The World Health Organization Disability Assessment Schedule 2.0 short form. HDRS: Hamilton Depression Rating Scale 17. HARS: Hamilton Anxiety Rating Scale. GAF-F: Global Assessment of Functioning – Function. GAF-S: Global Assessment of Functioning – Symptoms. HTQ: Harvard Trauma Questionnaire. HSCL: Hopkins Symptom Check List 25. WHO-5: The World Health Organization-Five Well-Being Index. SDS: Sheehan Disability Scale. PMLD: Post-migration Living Difficulties Check List. CHAI: Consumer Health Activation Index. EQ-5D-5L: EuroQol 5-Dimensions 5-Levels. | | | | | | | |

### Table S4. Completer analysis of group differences in outcomes (*n* = 195); ANCOVA/linear regression adjusted for baseline scores with multiple imputation.

|  | **Integrated Care** Mean diff | **TAU**  Mean diff | Group  diff | (95% CI) | *p*-value |
| --- | --- | --- | --- | --- | --- |
| WHODAS | 0.09 | 0.27 | 0.12 | (-2.85 to 3.20) | 0.91 |
| HDRS | 0.54 | 1.05 | 0.51 | (-1.84 to 2.85) | 0.67 |
| HARS | 1.86 | 2.59 | 0.73 | (-3.09 to 4.54) | 0.71 |
| GAF-F | 2.68 | 3.53 | 0.86 | (-1.77 to 3.48) | 0.52 |
| GAF-S | 2.69 | 3.48 | 0.79 | (-1.89 to 3.48) | 0.56 |
| HTQ | -0.17 | -0.17 | 0.00 | (-0.18 to 0.17) | 0.98 |
| HSCL | -0.22 | -0.12 | 0.10 | (-0.09 to 0.29) | 0.28 |
| WHO-5 | 6.52 | 4.36 | -2.16 | (-9.13 to 4.82) | 0.54 |
| SDS | -1.08 | -1.39 | -0.31 | (-2.61 to 2.00) | 0.79 |
| PMLD | -0.65 | -0.31 | 0.34 | (-0.81 to 1.50) | 0.56 |
| CHAI | -1.37 | 1.43 | 2.81 | (-2.96 to 8.57) | 0.34 |
| EQ-5D-5L | 0.03 | -0.01 | -0.04 | (-0.16 to 0.09) | 0.57 |
| TAU: Treatment as usual. Diff.: Difference. CI: Confidence interval. WHODAS: The World Health Organization Disability Assessment Schedule 2.0 short form. HDRS: Hamilton Depression Rating Scale 17. HARS: Hamilton Anxiety Rating Scale. GAF-F: Global Assessment of Functioning – Function. GAF-S: Global Assessment of Functioning – Symptoms. HTQ: Harvard Trauma Questionnaire. HSCL: Hopkins Symptom Check List 25. WHO-5: The World Health Organization-Five Well-Being Index. SDS: Sheehan Disability Scale. PMLD: Post-migration Living Difficulties Check List. CHAI: Consumer Health Activation Index. EQ-5D-5L: EuroQol 5-Dimensions 5-Levels. | | | | | |

### Table S5. Intention-to-treat analysis of group differences in outcomes (*n* = 195); ANCOVA/linear regression with multiple imputation, adjusted for baseline scores and years of psychiatric symptoms.

|  | **Integrated care** Mean diff | **TAU**  Mean diff | Group  diff | (95% CI) | *p*-value |
| --- | --- | --- | --- | --- | --- |
| WHODAS | 0.05 | 0.39 | 0.44 | (-2.33 to 3.22) | 0.75 |
| HDRS | 0.20 | 0.52 | 0.33 | (-1.98 to 2.63) | 0.78 |
| HARS | 1.05 | 2.27 | 1.21 | (-2.46 to 4.89) | 0.52 |
| GAF-F | 2.62 | 2.46 | -0.16 | (-2.71 to 2.38) | 0.90 |
| GAF-S | 2.93 | 2.73 | -0.19 | (-2.80 to 2.41) | 0.89 |
| HTQ | -0.19 | -0.15 | 0.04 | (-0.13 to 0.21) | 0.65 |
| HSCL | -0.24 | -0.03 | 0.21 | (0.03 to 0.40) | 0.025 |
| WHO-5 | 8.00 | 4.26 | -3.73 | (-10.63 to 3.16) | 0.29 |
| SDS | -1.42 | -0.63 | 0.78 | (-1.44 to 3.00) | 0.49 |
| PMLD | -0.85 | -0.14 | 0.72 | (-0.41 to 1.84) | 0.21 |
| CHAI | 0.97 | 0.92 | -0.05 | (-5.81 to 5.70) | 0.99 |
| EQ-5D-5L | 0.05 | -0.07 | -0.12 | (-0.24 to 0.00) | 0.044 |
| TAU: Treatment as usual. Diff.: Difference. CI: Confidence interval. WHODAS: The World Health Organization Disability Assessment Schedule 2.0 short form. HDRS: Hamilton Depression Rating Scale 17. HARS: Hamilton Anxiety Rating Scale. GAF-F: Global Assessment of Functioning – Function. GAF-S: Global Assessment of Functioning – Symptoms. HTQ: Harvard Trauma Questionnaire. HSCL: Hopkins Symptom Check List 25. WHO-5: The World Health Organization-Five Well-Being Index. SDS: Sheehan Disability Scale. PMLD: Post-migration Living Difficulties Check List. CHAI: Consumer Health Activation Index. EQ-5D-5L: EuroQol 5-Dimensions 5-Levels. | | | | | |

### Table S6. Observed case analysis of group differences in outcomes; ANCOVA/linear regression adjusted for baseline scores.

|  | **Integrated care** Mean diff | **TAU**  Mean diff | Group  diff | (95% CI) | *p*-value |
| --- | --- | --- | --- | --- | --- |
| WHODAS (*n*=154) | -0.09 | 0.41 | 0.50 | (-2.22 to 3.23) | 0.72 |
| HDRS (*n*=144) | 0.34 | 0.51 | 0.17 | (-2.15 to 2.48) | 0.89 |
| HARS (*n*=144) | 1.36 | 1.87 | 0.51 | (-3.21 to 4.22) | 0.79 |
| GAF-F (*n*=192) | 2.47 | 2.62 | 0.16 | (-2.33 to 2.64) | 0.90 |
| GAF-S (*n*=191) | 2.73 | 2.83 | 0.09 | (-2.44 to 2.63) | 0.94 |
| HTQ (*n*=161) | -0.18 | -0.15 | 0.03 | (-0.13 to 0.19) | 0.69 |
| HSCL (*n*=160) | -0.23 | -0.06 | 0.17 | (-0.01 to 0.34) | 0.064 |
| WHO-5 (*n*=155) | 7.39 | 3.83 | -3.56 | (-10.13 to 3.00) | 0.29 |
| SDS (*n*=153) | -1.28 | -0.96 | 0.32 | (-1.90 to 2.54) | 0.78 |
| PMLD (*n*=139) | -0.72 | -0.15 | 0.57 | (-0.51 to 1.65) | 0.30 |
| CHAI (*n*=130) | 0.07 | 0.19 | 0.12 | (-5.10 to 5.34) | 0.96 |
| EQ-5D-5L (*n*=136) | 0.05 | -0.05 | -0.10 | (-0.22 to 0.03) | 0.12 |
| TAU: Treatment as usual. Diff.: Difference. CI: Confidence interval. WHODAS: The World Health Organization Disability Assessment Schedule 2.0 short form. HDRS: Hamilton Depression Rating Scale 17. HARS: Hamilton Anxiety Rating Scale. GAF-F: Global Assessment of Functioning – Function. GAF-S: Global Assessment of Functioning – Symptoms. HTQ: Harvard Trauma Questionnaire. HSCL: Hopkins Symptom Check List 25. WHO-5: The World Health Organization-Five Well-Being Index. SDS: Sheehan Disability Scale. PMLD: Post-migration Living Difficulties Check List. CHAI: Consumer Health Activation Index. EQ-5D-5L: EuroQol 5-Dimensions 5-Levels. | | | | | |

### Table S7. Low-value scenario of group differences in outcomes (*n* = 195); intention-to-treat sample with missing outcomes imputed as mean –2 SD, adjusted for baseline scores.

|  | **Integrated care** Mean diff | **TAU**  Mean diff | Group  diff | (95% CI) | *p*-value |
| --- | --- | --- | --- | --- | --- |
| WHODAS | -2.11 | -3.41 | -1.30 | (-4.48 to 1.89) | 0.42 |
| HDRS | -2.31 | -2.59 | -0.27 | (-2.81 to 2.26) | 0.83 |
| HARS | -2.77 | -2.75 | 0.02 | (-4.00 to 4.02) | 0.99 |
| GAF-F | 2.45 | 1.97 | -0.48 | (-3.04 to 2.07) | 0.71 |
| GAF-S | 2.75 | 2.10 | -0.65 | (-3.24 to 1.94) | 0.62 |
| HTQ | -0.29 | -0.32 | -0.04 | (-0.21 to 0.14) | 0.68 |
| HSCL | -0.33 | -0.25 | 0.07 | (-0.12 to 0.26) | 0.46 |
| WHO-5 | 1.01 | -2.72 | -3.74 | (-10.72 to 3.24) | 0.29 |
| SDS | -2.29 | -2.51 | -0.21 | (-2.52 to 2.09) | 0.86 |
| PMLD | -2.67 | -2.20 | 0.47 | (-0.88 to 1.82) | 0.49 |
| CHAI | -8.74 | -8.63 | 0.11 | (-5.97 to 6.20) | 0.97 |
| EQ-5D-5L | -0.05 | -0.10 | -0.05 | (-0.19 to 0.08) | 0.44 |
| TAU: Treatment as usual. Diff.: Difference. CI: Confidence interval. WHODAS: The World Health Organization Disability Assessment Schedule 2.0 short form. HDRS: Hamilton Depression Rating Scale 17. HARS: Hamilton Anxiety Rating Scale. GAF-F: Global Assessment of Functioning – Function. GAF-S: Global Assessment of Functioning – Symptoms. HTQ: Harvard Trauma Questionnaire. HSCL: Hopkins Symptom Check List 25. WHO-5: The World Health Organization-Five Well-Being Index. SDS: Sheehan Disability Scale. PMLD: Post-migration Living Difficulties Check List. CHAI: Consumer Health Activation Index. EQ-5D-5L: EuroQol 5-Dimensions 5-Levels. | | | | | |

### Table S8. High-value scenario of group differences in outcomes (*n* = 195); intention-to-treat sample with missing outcomes imputed as mean +2 SD, adjusted for baseline scores.

|  | **Integrated care** Mean diff | **TAU**  Mean diff | Group  diff | (95% CI) | *p*-value |
| --- | --- | --- | --- | --- | --- |
| WHODAS | 3.12 | 4.80 | 1.68 | (-1.66 to 5.02) | 0.32 |
| HDRS | 2.69 | 3.32 | 0.63 | (-1.87 to 3.13) | 0.62 |
| HARS | 5.01 | 6.52 | 1.51 | (-2.46 to 5.48) | 0.45 |
| GAF-F | 2.45 | 3.16 | 0.71 | (-1.81 to 3.24) | 0.58 |
| GAF-S | 2.76 | 3.66 | 0.90 | (-1.71 to 3.52) | 0.50 |
| HTQ | 0.01 | 0.05 | 0.04 | (-0.15 to 0.22) | 0.69 |
| HSCL | -0.02 | 0.13 | 0.15 | (-0.05 to 0.35) | 0.13 |
| WHO-5 | 11.16 | 10.33 | -0.83 | (-7.79 to 6.12) | 0.81 |
| SDS | 0.69 | 0.89 | 0.20 | (-2.12 to 2.53) | 0.86 |
| PMLD | 1.28 | 1.88 | 0.60 | (-0.71 to 1.90) | 0.37 |
| CHAI | 10.57 | 12.00 | 1.43 | (-5.15 to 8.01) | 0.67 |
| EQ-5D-5L | 0.10 | 0.02 | -0.08 | (-0.22 to 0.05) | 0.23 |
| TAU: Treatment as usual. Diff.: Difference. CI: Confidence interval. WHODAS: The World Health Organization Disability Assessment Schedule 2.0 short form. HDRS: Hamilton Depression Rating Scale 17. HARS: Hamilton Anxiety Rating Scale. GAF-F: Global Assessment of Functioning – Function. GAF-S: Global Assessment of Functioning – Symptoms. HTQ: Harvard Trauma Questionnaire. HSCL: Hopkins Symptom Check List 25. WHO-5: The World Health Organization-Five Well-Being Index. SDS: Sheehan Disability Scale. PMLD: Post-migration Living Difficulties Check List. CHAI: Consumer Health Activation Index. EQ-5D-5L: EuroQol 5-Dimensions 5-Levels. | | | | | |

### Table S9: Group differences in outcomes without outliers (*n* = 195); intention-to-treat sample with multiple imputation, excluding values ±2 SD, adjusted for baseline scores.

|  | **Integrated care** Mean diff. | **TAU**  Mean diff. | Group  diff. | (95% CI) | *p*-value |
| --- | --- | --- | --- | --- | --- |
| WHODAS (n=188) | 0.50 | 0.86 | 0.36 | (-1.99 to 2.71) | 0.76 |
| HDRS (n=187) | 1.17 | 1.19 | 0.02 | (-1.94 to 1.98) | 0.98 |
| HARS (n=188) | 2.01 | 2.83 | 0.82 | (-2.20 to 3.84) | 0.59 |
| GAF-F (n=188) | 1.83 | 2.71 | 0.88 | (-1.14 to 2.90) | 0.39 |
| GAF-S (n=188) | 2.11 | 3.47 | 1.36 | (-0.51 to 2.23) | 0.15 |
| HTQ (n=187) | -0.13 | -0.06 | 0.07 | (-0.06 to 0.20) | 0.27 |
| HSCL (n=186) | -0.16 | 0.03 | 0.19 | (0.04 to 0.34) | 0.015 |
| WHO-5 (n=190) | 6.06 | 0.68 | -5.38 | (-11.00 to 0.24) | 0.061 |
| SDS (n=183) | -0.59 | -0.22 | 0.37 | (-1.35 to 2.09) | 0.67 |
| PMLD (n=187) | -0.80 | -0.51 | 0.29 | (-0.70 to 1.28) | 0.56 |
| CHAI (n=189) | 1.94 | -0.43 | -2.38 | (-7.34 to 2.59) | 0.35 |
| EQ-5D-5L (n=187) | 0.02 | -0.07 | -0.09 | (-0.20 to 0.02) | 0.098 |
| TAU: Treatment as usual. Diff.: Difference. CI: Confidence interval. WHODAS: The World Health Organization Disability Assessment Schedule 2.0 short form. HDRS: Hamilton Depression Rating Scale 17. HARS: Hamilton Anxiety Rating Scale. GAF-F: Global Assessment of Functioning – Function. GAF-S: Global Assessment of Functioning – Symptoms. HTQ: Harvard Trauma Questionnaire. HSCL: Hopkins Symptom Check List 25. WHO-5: The World Health Organization-Five Well-Being Index. SDS: Sheehan Disability Scale. PMLD: Post-migration Living Difficulties Check List. CHAI: Consumer Health Activation Index. EQ-5D-5L: EuroQol 5-Dimensions 5-Levels. | | | | | |

### Table S10. Summary of results from assumption testing.

|  | **Normal distribution** | | | **Homoscedasticity** | | **Outliers** | |
| --- | --- | --- | --- | --- | --- | --- | --- |
|  | Shapiro-Wilken  Before log  *p*-value | Shapiro-Wilken  After log  *p*-value | Mann-Whitney  *p*-value | Levene’s  *p*-value | Breusch-Pagan  *p*-value | +/- 2 SD  No. | Cook’s D  No. |
| WHODAS | 0.055 | - | - | 0.51 | <0.001 | 7 | 18 |
| HDRS | 0.001 | <0.001 | 1.00 | 0.53 | <0.001 | 8 | 9 |
| HARS | 0.002 | <0.001 | 0.79 | 0.56 | <0.001 | 7 | 7 |
| GAF-F | <0.001 | 0.55 | 0.36 | 0.080 | 0.002 | 7 | 14 |
| GAF-S | <0.001 | <0.001 | 0.10 | 0.29 | 0.64 | 7 | 13 |
| HTQ | <0.001 | <0.001 | 0.55 | 0.70 | <0.001 | 8 | 11 |
| HSCL | <0.001 | <0.001 | 0.051 | 0.71 | <0.001 | 9 | 11 |
| WHO-5 | <0.001 | 0.62 | 0.30 | 0.40 | 0.072 | 5 | 14 |
| SDS | <0.001 | <0.001 | 0.64 | 0.61 | <0.001 | 12 | 11 |
| PMLD | 0.46 | - | - | 0.20 | 0.49 | 8 | 15 |
| CHAI | 0.70 | - | - | 0.93 | 0.74 | 6 | 15 |
| EQ-5D-5L | 0.098 | - | - | 0.91 | 0.052 | 8 | 9 |
| SD: Standard deviation.. WHODAS: The World Health Organization Disability Assessment Schedule 2.0 short form. HDRS: Hamilton Depression Rating Scale 17. HARS: Hamilton Anxiety Rating Scale. GAF-F: Global Assessment of Functioning – Function. GAF-S: Global Assessment of Functioning – Symptoms. HTQ: Harvard Trauma Questionnaire. HSCL: Hopkins Symptom Check List 25. WHO-5: The World Health Organization-Five Well-Being Index. SDS: Sheehan Disability Scale. PMLD: Post-migration Living Difficulties Check List. CHAI: Consumer Health Activation Index. EQ-5D-5L: EuroQol 5-Dimensions 5-Levels. | | | | | | | |

### Table S11: Number of patients included per year.

|  | **2020** | **2021** | **2022** | **2023** | **2024** |
| --- | --- | --- | --- | --- | --- |
| Patients included | 45 | 49 | 44 | 41 | 16 |
| *WHO defines the COVID-19 pandemic as an international emergency from 2020 to 2023. In Denmark, COVID-19 restrictions were implemented from March 2020 to February 2022, and thus, pandemic years in the subsample analysis are defined as patients included in 2020 and 2021.* | | | | | |

### Table S12. Comparison of outcomes between intervention groups in the subsample included during non-pandemic years (2022–2024); intention-to-treat sample (*n* = 101), ANCOVA/linear regression adjusted for baseline scores with multiple imputation.

|  | **Integrated care** | | | **TAU** | | |  | | |
| --- | --- | --- | --- | --- | --- | --- | --- | --- | --- |
|  | Baseline score | End score | Diff. score | Baseline score | End score | Diff. score | Group  diff | (95% CI) | *p*-value |
| WHODAS | 29.97 | 29.98 | 0.08 | 29.47 | 30.29 | 0.76 | 0.69 | (-3.17 to 4.54) | 0.72 |
| HDRS | 23.82 | 24.10 | 0.20 | 24.44 | 24.92 | 0.57 | 0.38 | (-2.84 to 3.60) | 0.82 |
| HARS | 34.87 | 35.23 | 0.43 | 34.45 | 37.04 | 2.52 | 2.09 | (-3.05 to 7.22) | 0.42 |
| GAF-F | 47.08 | 50.16 | 3.06 | 47.16 | 48.26 | 1.13 | -1.93 | (-5.30 to 1.44) | 0.26 |
| GAF-S | 49.27 | 52.67 | 3.68 | 48.04 | 50.53 | 2.18 | -1.49 | (-5.07 to 2.09) | 0.41 |
| HTQ | 3.09 | 2.91 | -0.18 | 3.13 | 2.93 | -0.20 | -0.02 | (-0.28 to 0.25) | 0.91 |
| HSCL | 2.98 | 2.67 | -0.31 | 3.00 | 2.90 | -0.10 | 0.22 | (-0.05 to 0.48) | 0.10 |
| WHO-5 | 21.63 | 27.07 | 7.63 | 14.00 | 20.79 | 4.37 | -3.26 | (-13.52 to 7.00) | 0.53 |
| SDS | 23.33 | 22.12 | -0.53 | 21.21 | 23.04 | 1.08 | 1.61 | (-1.68 to 4.90) | 0.33 |
| PMLD | 7.24 | 6.79 | -0.52 | 7.80 | 7.37 | -0.36 | 0.16 | (-1.39 to 1.70) | 0.84 |
| CHAI | 58.43 | 60.83 | 2.82 | 56.56 | 55.51 | -1.51 | -4.33 | (-11.93 to 3.27) | 0.26 |
| EQ-5D-5L | 0.13 | 0.13 | 0.00 | 0.14 | 0.06 | -0.08 | -0.08 | (-0.27 to 0.12) | 0.43 |
| TAU: Treatment as usual. Diff.: Difference. CI: Confidence interval. WHODAS: The World Health Organization Disability Assessment Schedule 2.0 short form. HDRS: Hamilton Depression Rating Scale 17. HARS: Hamilton Anxiety Rating Scale. GAF-F: Global Assessment of Functioning – Function. GAF-S: Global Assessment of Functioning – Symptoms. HTQ: Harvard Trauma Questionnaire. HSCL: Hopkins Symptom Check List 25. WHO-5: The World Health Organization-Five Well-Being Index. SDS: Sheehan Disability Scale. PMLD: Post-migration Living Difficulties Check List. CHAI: Consumer Health Activation Index. EQ-5D-5L: EuroQol 5-Dimensions 5-Levels. | | | | | | | | | |

### Table S13. Comparison of outcomes in the integrated care group during pandemic (2020–2021) vs non-pandemic years (2022–2024); intention-to-treat sample (*n* = 98), ANCOVA/linear regression adjusted for baseline scores with multiple imputation.

|  | **Pandemic years** | | | **Non-pandemic years** | | |  | | |
| --- | --- | --- | --- | --- | --- | --- | --- | --- | --- |
|  | Baseline score | End score | Diff. score | Baseline score | End score | Diff. score | Group  diff | (95% CI) | *p*-value |
| WHODAS | 30.73 | 31.13 | 0.52 | 30.03 | 29.83 | -0.30 | 0.82 | (-3.29 to 4.92) | 0.69 |
| HDRS | 22.67 | 22.77 | -0.19 | 23.94 | 24.09 | 0.40 | -0.59 | (-4.18 to 3.01) | 0.74 |
| HARS | 31.63 | 33.78 | 1.30 | 34.83 | 35.07 | 0.96 | 0.34 | (-4.65 to 5.33) | 0.89 |
| GAF-F | 48.50 | 50.04 | 1.75 | 47.11 | 50.27 | 2.99 | -1.24 | (-4.65 to 2.18) | 0.47 |
| GAF-S | 49.72 | 51.18 | 1.56 | 49.26 | 52.79 | 3.45 | -1.88 | (-5.77 to 2.01) | 0.34 |
| HTQ | 3.10 | 2.94 | -0.17 | 3.09 | 2.92 | -0.15 | 0.02 | (-0.23 to 0.26) | 0.88 |
| HSCL | 2.97 | 2.85 | -0.12 | 2.98 | 2.66 | -0.31 | 0.20 | (-0.07 to 0.46) | 0.14 |
| WHO-5 | 14.77 | 22.77 | 6.35 | 21.43 | 26.97 | 6.94 | -0.59 | (-9.54 to 8.36) | 0.90 |
| SDS | 22.52 | 21.31 | -1.52 | 23.37 | 22.95 | -1.05 | -0.47 | (-3.71 to 2.77) | 0.77 |
| PMLD | 7.21 | 6.10 | -1.11 | 7.26 | 6.82 | -0.44 | -0.67 | (-2.34 to 0.99) | 0.42 |
| CHAI | 58.99 | 58.01 | -0.92 | 58.72 | 60.93 | 2.17 | -3.09 | (-12.00 to 5.81) | 0.49 |
| EQ-5D-5L | 0.00 | 0.09 | 0.07 | 0.12 | 0.14 | 0.04 | 0.03 | (-0.16 to 0.22) | 0.75 |
| Diff.: Difference. CI: Confidence interval. WHODAS: The World Health Organization Disability Assessment Schedule 2.0 short form. HDRS: Hamilton Depression Rating Scale 17. HARS: Hamilton Anxiety Rating Scale. GAF-F: Global Assessment of Functioning – Function. GAF-S: Global Assessment of Functioning – Symptoms. HTQ: Harvard Trauma Questionnaire. HSCL: Hopkins Symptom Check List 25. WHO-5: The World Health Organization-Five Well-Being Index. SDS: Sheehan Disability Scale. PMLD: Post-migration Living Difficulties Check List. CHAI: Consumer Health Activation Index. EQ-5D-5L: EuroQol 5-Dimensions 5-Levels. | | | | | | | | | |

### Table S14. Comparison of outcomes in TAU group during pandemic (2020–2021) vs non-pandemic years (2022–2024); intention-to-treat sample (*n* = 98), ANCOVA/linear regression adjusted for baseline scores with multiple imputation.

|  | **Pandemic years** | | | **Non-pandemic years** | | |  | | |
| --- | --- | --- | --- | --- | --- | --- | --- | --- | --- |
|  | Baseline score | End score | Diff. score | Baseline score | End score | Diff. score | Group  diff | (95% CI) | *p*-value |
| WHODAS | 29.45 | 29.08 | -0.37 | 29.45 | 30.60 | 1.15 | -1.52 | (-5.46 to 2.42) | 0.44 |
| HDRS | 22.60 | 22.17 | -0.60 | 24.24 | 24.67 | 0.61 | -1.22 | (-4.66 to 2.23) | 0.48 |
| HARS | 31.53 | 31.91 | 0.05 | 34.33 | 36.47 | 2.48 | -2.42 | (-8.93 to 4.08) | 0.46 |
| GAF-F | 48.13 | 52.12 | 4.24 | 47.05 | 48.39 | 1.07 | 3.17 | (-0.60 to 6.94) | 0.098 |
| GAF-S | 49.20 | 52.66 | 3.72 | 48.03 | 50.64 | 2.35 | 1.37 | (-2.09 to 4.84) | 0.43 |
| HTQ | 3.12 | 3.00 | -0.12 | 3.12 | 2.93 | -0.19 | 0.07 | (-0.16 to 0.31) | 0.54 |
| HSCL | 2.96 | 2.92 | -0.04 | 2.98 | 2.88 | -0.10 | 0.05 | (-0.22 to 0.32) | 0.70 |
| WHO-5 | 15.65 | 18.55 | 3.32 | 14.14 | 21.01 | 6.46 | -3.14 | (-13.14 to 6.87) | 0.53 |
| SDS | 23.10 | 19.97 | -2.66 | 21.24 | 23.15 | 1.43 | -4.09 | (-7.35 to -0.83) | 0.015 |
| PMLD | 7.24 | 7.37 | 0.04 | 7.76 | 7.37 | -0.29 | 0.32 | (-1.46 to 2.11) | 0.72 |
| CHAI | 57.20 | 60.87 | 3.73 | 56.77 | 55.24 | -1.59 | 5.31 | (-4.04 to 14.67) | 0.26 |
| EQ-5D-5L | 0.07 | 0.02 | -0.07 | 0.15 | 0.06 | -0.07 | 0.00 | (-0.19 to 0.18) | 0.96 |
| TAU: Treatment as usual. Diff.: Difference. CI: Confidence interval. WHODAS: The World Health Organization Disability Assessment Schedule 2.0 short form. HDRS: Hamilton Depression Rating Scale 17. HARS: Hamilton Anxiety Rating Scale. GAF-F: Global Assessment of Functioning – Function. GAF-S: Global Assessment of Functioning – Symptoms. HTQ: Harvard Trauma Questionnaire. HSCL: Hopkins Symptom Check List 25. WHO-5: The World Health Organization-Five Well-Being Index. SDS: Sheehan Disability Scale. PMLD: Post-migration Living Difficulties Check List. CHAI: Consumer Health Activation Index. EQ-5D-5L: EuroQol 5-Dimensions 5-Levels. | | | | | | | | | |

### Table S15. Available data for all outcomes at each time point.

|  | **Pre-treatment** | **Post-treatment** |  |
| --- | --- | --- | --- |
| WHODAS | 187 | 157 |  |
| HDRS | 178 | 149 |  |
| HARS | 178 | 149 |  |
| GAF-F | 195 | 192 |  |
| GAF-S | 195 | 191 |  |
| HTQ | 188 | 162 |  |
| HSCL | 187 | 162 |  |
| WHO-5 | 183 | 160 |  |
| SDS | 179 | 159 |  |
| PMLD | 188 | 141 |  |
| CHAI | 185 | 135 |  |
| EQ-5D-5L | 167 | 154 |  |
| WHODAS: The World Health Organization Disability Assessment Schedule 2.0 short form. HDRS: Hamilton Depression Rating Scale 17. HARS: Hamilton Anxiety Rating Scale. GAF-F: Global Assessment of Functioning – Function. GAF-S: Global Assessment of Functioning – Symptoms. HTQ: Harvard Trauma Questionnaire. HSCL: Hopkins Symptom Check List 25. WHO-5: The World Health Organization-Five Well-Being Index. SDS: Sheehan Disability Scale. PMLD: Post-migration Living Difficulties Check List. CHAI: Consumer Health Activation Index. EQ-5D-5L: EuroQol 5-Dimensions 5-Levels. | | | |
